# Variations in microanatomy of the human modiolus require individualized cochlear implantation

**DOI:** 10.1038/s41598-022-08731-x

**Published:** 2022-03-23

**Authors:** Markus Pietsch, Daniel Schurzig, Rolf Salcher, Athanasia Warnecke, Peter Erfurt, Thomas Lenarz, Andrej Kral

**Affiliations:** 1 Department of Otolaryngology, Hannover Medical School, Hannover, Germany; 2 Department of Otolaryngology, Helios Clinic Hildesheim, Hildesheim, Germany; 3 Department of Experimental Otology, Hannover Medical School, Hannover, Germany; 4 MED-EL Research Center, Hannover, Germany; 5 Department of Biomedical Sciences, Faculty of Medicine and Health Sciences, Macquarie University, Sydney, Australia

**Keywords:** Anatomy, Medical research

## Abstract

Cochlear variability is of key importance for the clinical use of cochlear implants, the most successful neuroprosthetic device that is surgically placed into the cochlear scala tympani. Despite extensive literature on human cochlear variability, few information is available on the variability of the modiolar wall. In the present study, we analyzed 108 corrosion casts, 95 clinical cone beam computer tomographies (CTs) and 15 µCTs of human cochleae and observed modiolar variability of similar and larger extent than the lateral wall variability. Lateral wall measures correlated with modiolar wall measures significantly. ~ 49% of the variability had a common cause. Based on these data we developed a model of the modiolar wall variations and related the model to the design of cochlear implants aimed for perimodiolar locations. The data demonstrate that both the insertion limits relevant for lateral wall damage (approximate range of 4–9 mm) as well as the dimensions required for optimal perimodiolar placement of the electrode (the point of release from the straightener; approximate range of 2–5mm) are highly interindividually variable. The data demonstrate that tip fold-overs of preformed implants likely result from the morphology of the modiolus (with radius changing from base to apex), and that optimal cochlear implantation of perimodiolar arrays cannot be guaranteed without an individualized surgical technique.

## Introduction

The shape of the human cochlea has an intriguing three-dimensional geometry that is reminiscent of the shell of a nautilus, which remarkably fits to a logarithmic spiral^1–3^. A relation of the cochlear form to an acoustic function has been proposed^4^, but the interindividual variability of the human cochlea^5–10^ was inconsistent with this proposal^8^ and suggested that spatial constraints in the temporal bone define the cochlear shape^11,12^. The exact shape and its variation were not compatible with a nautilus-like logarithmic spiral, but rather fits to a more complex polynomial spiral (Ref.^8^, comp.^13^). Thus, the interindividual variability in the microanatomy of the human cochlea is substantial and the details have a complex geometry.

Human cochlear variability is of key importance for cochlear implantation. Implantation trauma and postoperative hearing outcomes are dependent on the mutual relation of cochlear size and the implant electrode^14–17^. Furthermore, variability in the vertical trajectory of the cochlear implant array can cause damage to the basilar membrane^7,18,19^. In these studies the vertical profile and the dimension of the scala tympani was less variable near the modiolus. Such an observation would favor perimodiolar electrodes^20–22^, particularly since reduced distance to the modiolus may reduce channel interactions and reduce thresholds^23–25^. However, implantation trauma may be a serious complication^26–29^. Damage to the modiolus leads to loss of spiral ganglion cells^30^ and may represent a route for infections into the intrathecal space^31^. Furthermore, perimodiolar placements require preformed electrode arrays^21,24^. These cannot be implanted in their precurved form, and even using a positioner (straightener or stylet) that straightens their form for implantation still involves the risk of a fold-over of the electrode array once it is released from the positioner^21,32–34^ or the risk of a scalar translocation^35^. In fact, a recent retrospective study on 1722 cochlear implantations reported that dependent on the electrode array that was implanted, tip fold-over rates may go up to 10.5%^33^. This is problematic since identification of these incidents typically involves additional, intraoperative imaging, entailing additional radiation exposure and surgery time. Alternatively, experimental software can be used to identify tip fold-overs, but this again involves additional surgery time. The preoperative identification of anatomies where tip fold-overs are more likely to occur would hence be highly beneficial. Up to now, no detailed analysis of the relation between the electrode array and the modiolus and its interindividual variability has been published yet. Knowledge on cochlear anatomy and its individual variations is of key importance for cochlear implantations of perimodiolar arrays.

Furthermore, it has been suggested that cochlear variability is due to the facial nerve, jugular vein, internal carotid and the tensor tympani muscle that are in close proximity of the cochlea and that form before the cochlear scalae^8^. The modiolus is ontogenetically formed before cochlear scalae^36^. Therefore, studying the modiolus in its interindividual variability would provide information whether developmentally, variability is established during cochlear spaces formation, or before their appearance. The latter would indicate that the formation of neural structures (that are the early structural basis of the modiolar geometry) is responsible for a substantial amount of cochlear variability.

The goal of the present study was to evaluate the variability of modiolar parts of the cochlea and compare it to the variations observed with measures obtained from the lateral wall. Three groups of specimen were compared: corrosion casts^8^, micro computer tomography (µCT) datasets^37^ and clinical measurements obtained with cone beam computer tomography (CT) in a clinical setting^38^. The data show that the variability in cochlear microanatomy is similar in modiolar and lateral portions of the cochlea. The data presented allows for conclusions on current design issues of perimodiolar arrays.

## Materials and methods

**Figure 1:**
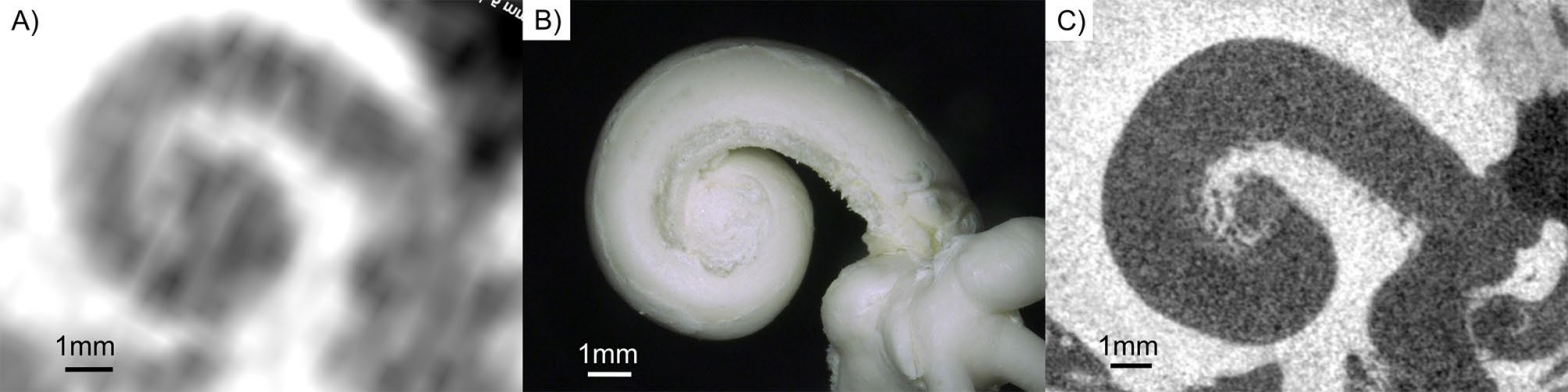
Imaging of the cochlea using the three methods used in the present study: (**A**) Cone beam computer tomography (CBCT); (**B**) Corrosion Cast; (**C**) micro computer tomography (µCT). The different methods differ in resolution and details, with corrosion casts and µCTs providing better resolution than CBCT.

Three different datasets of human cochlear anatomy were used in the present study: cone beam CT (CBCT) obtained in clinical setting before cochlear implantation (Fig. 1A), corrosion casts from donors (Fig. 1B) and micro-CTs (µCTs) from donors (Fig. 1C). While CBCT can be obtained in living human subjects, both corrosion casts and µCT are obtained from cadaver temporal bones. All methods were performed in accordance with the relevant guidelines and regulations.

### CBCT measurements (“clinical CT”)

The method of CBCT imaging and analysis and the dataset have been described in detail previously^38–40^; here we reuse these data. In brief, a total of 95 patients (51 female, 44 male) with cochlear implants were included in the analysis. The age of the patients ranged between 2 and 83 years (mean 54.3 years). All patients were treated at the Department of Otorhinolaryngology—Head and Neck Surgery of Hanover Medical School. Clinical CT images were anonymized. The institutional ethics committee at Hannover Medical School approved the use of anonymized imaging data obtained within the clinical routine. Segmentations were performed in clinical CBCT datasets acquired prior to surgery. CBCT datasets were generated using the Xoran XCAT (125 kVp, 7 mA) resulting in an isotropic voxel size of 0.3 mm or the Morita 3D Accuitomo 170 set to an isotropic voxel size of 0.08 mm.

These clinical scans are part of the clinical routine at the Hannover Medical School to preoperatively evaluate the condition of the cochlea and postoperatively confirm correct intracochlear array placement. All segmentations of the cochlear modiolar wall in preoperative CBCT data were performed with the software tool OsiriX MD (version 2.5.1 64bit, Pixmeo SARL, Switzerland) according to previous studies^39–42^. For a standardized view, window width was set to 4600 Hounsfield Units (HU) and window leveling was set to 1095 HU. The modiolar wall was measured along the A and B axis according to the previously accepted guidelines^43^.

### µCT

The method used for 15 µCTs has been described in detail previously^40^. In brief, 15 anonymized µCT data sets generated by a SCANCO MicroCT 100 (version 1.1, SCANCO Medical AG, Switzerland) were processed. The scans were performed at 70 kVp and 114 or 88 µA with AI05 or Cu01 filtering, resulting in a voxel size of 10 × 10 × 10 µm. The data sets were loaded into a custom software tool specifically developed for accurate segmentation of the cochlea. The utilized custom-made segmentation tool was programmed in C++^44^ with the goal to maximize the accuracy of the segmented cochlear structures. The resulting segmentation data points were then processed and converted within three main steps, all of which were performed in MatLab (version R2018a, The MathWorks Inc., USA) according to Ref.^40^. The cochlear lumina including the modiolus were segmented with an angular step width of 22.5°, which was proven to be sufficiently small to serve as the foundation of convergence studies during data evaluation. Correspondingly, also here A and B measurements were performed according to Ref.^43^.

### Corrosion casts

The method used for 108 corrosion casts of human cochleae (59 left, 49 right) has been described in detail previously^8^. In brief, very high-resolution imaging (12 µm/pixel) in precise reproducible cross-hair-laser-assisted positioned views (according to the Consensus Cochlear Coordinate System/CCCS^43^) of corrosion casts from the Hanover Human Cochlea Database were studied. Measurements of distances, angles and areas were performed with the microscope manufacturers’ analysis software in maximal magnification (Keyence VHX-600). Measurement of cochlear length was performed with ImageJ software (Image Processing and Analysis in Java, freeware, available at http://rsbweb.nih.gov/ij/), which was calibrated for the pixel resolution. 120 measurement points in each of the 108 cochleae resulted in 11,324 total measurements due to 818 missing values, mainly because the measurement point exceeded the given cochlea (e.g. measures at 990° were only available in cochleae that reached this angular length, in smaller cochleae these measurements were not available).

Five standardized aspects were recorded for each specimen:

1. *Top axial* view on the cochlea along the modiolar axis, a perpendicular line to the modiolar axis was aligned horizontally through the midpoint of the round window. This view is matching the ‘plane of rotation’ of the CCCS and is equivalent to the defined radiographic projection of the ‘Cochlear View’.
2. *Base axial* view on the cochlea, exact opposite view to top axial view.
3. *Lateral* “round window view” on the cochlea, perpendicular view from the vestibule on the modiolar axis, which is aligned horizontally through the midpoint of the round-window.
4. *Medial* “ascending spiral view” on the cochlea, exact opposite view to lateral.
5. *Ventral* “side view” on the cochlea, perpendicular view from ventral on the modiolar axis, which is aligned horizontally.

The present study was performed based on the base axial view.

**Figure 2:**
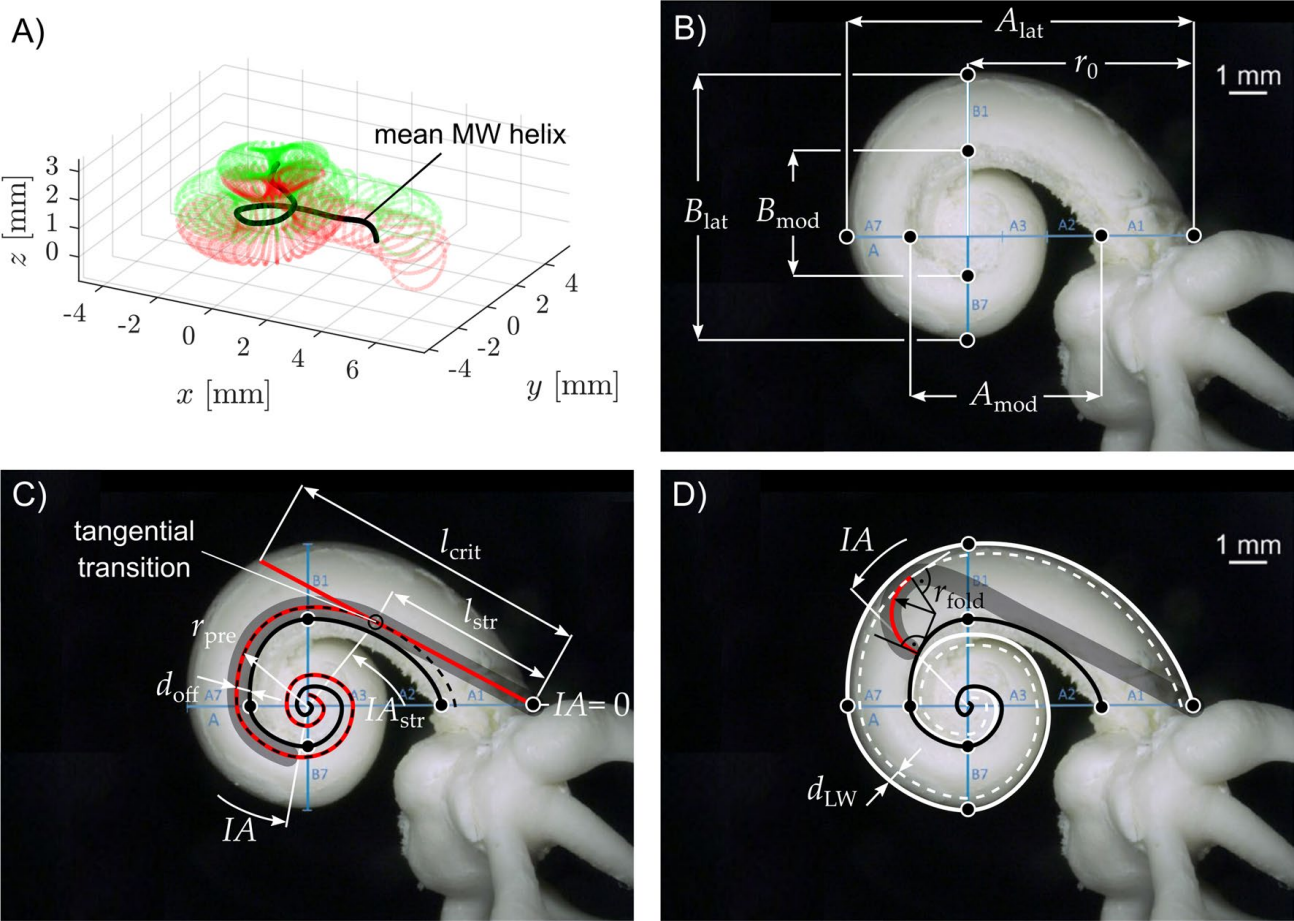
The methodological approach. (**A**) The average 3D profile of the cochlear MW extracted from the 15 µCT segmentations described in Ref.^37^. (**B**) Base axial view (see methods) at a left cochlea. Depiction of the cochlear dimensions A and B along the cochlear lateral (A_lat_, B_lat_) and modiolar wall as well as the distance r_0_ from the modiolar axis to the center of the round window. Please note that not all segments of the A and B axes are visible in this base axial view - for details see ref.^37^. (**C**) visualization of the computed insertion trajectory (in red) based on the individualized MW profile (solid black line) and distance d_off_ between MW and central axis of a perimodiolar array. l_str_ and IA_str_ describe the distance and insertion angle respectively after which straight part of the insertion trajectory ends. r_pre_ describes the curvature of the trajectory after the straight section. l_crit_ represents the distance at which the insertion of a straightened array would touch the lateral wall and potentially cause damage. (**D**) The computations of the critical radii (r_fold_) were based on the assumption that if the radius of the precurved implant is small enough for the tip to “stand up” inside the scala tympani, a tip fold-over becomes likely. For this reason, such hypothetical critical radius was computed depending on the different modiolar dimensions and different insertion angles (IAs). The minimal distance between the lateral wall and the central axis of an inserted array was denoted d_LW_.

The calculation of essential parameters of the present study (cf. Fig. 2B) based on the measurement values stated in Ref.^8^ was performed as follows:

- $${A}_{lat}={\sum }_{i=1}^{7}{A}_{i},$$
- $${B}_{lat}={\sum }_{i=1}^{7}{B}_{i},$$
- $${A}_{mod}={A}_{lat}-{A}_{1}-{A}_{7},$$
- $${B}_{mod}={B}_{lat}-{B}_{1}-{B}_{7},$$
- $${r}_{0}={\sum }_{i=1}^{4}{A}_{i}.$$

These values were compared to the ones derived in CT data and also used to scale average spiral models of the lateral and modiolar wall respectively, which is described in detail in the following subsection.

### Data analysis

Segmentation models from the 15 µCT datasets were used to create a mean 3D of the modiolar wall profile. First, the segmentation models of the 15 µCT datasets were averaged, yielding a mean representation of the human cochlea. A detailed description of the averaging procedure can be found in a previous study^45^. Based on this volumetric model the mean modiolar wall helix was subsequently extracted, as is depicted in Fig. 2A.

Individual cochlear diameter and width values for both the modiolar (*A*_*mod*_, *B*_*mod*_) and lateral wall (*A*_*lat*_, *B*_*lat*_) were determined at the point where the porous modiolar wall transformed to the smooth scala tympani portion (Fig. 2B). For this analysis, absolute values were compared, but additionally the values were normalized to the mean to assess the relative variance of the population. For this the values were normalized as

1 $$x_{norm} = \frac{{x_{1} - \overline{x} }}{{\overline{x} }}.$$

The A and B measures along the lateral and modiolar walls respectively were then used to create individualized 3D representations of the modiolar and lateral wall for the individual corrosion casts. This was performed using the regression-scaling (RS) model^46^ for the lateral wall with the input parameters *A*_*lat*_ and *B*_*lat.*_ Given that a regression scaling model is not available for the modiolar wall, the previously derived mean modiolar wall spiral was individualized using the ABH model^37^ with the individual input parameters *A*_*mod*_ and *B*_*mod*_. Note that since the RS model better mimics the individual height characteristics of the cochlear spiral, the height profile of the lateral wall profile was projected onto the modiolar wall. As depicted in Fig. 2C, these representations then allow for a model-based assessment of the relation between the cochlear insertion depth (metric and angular) to the distance from the modiolus *d*_off_. The model was based on the corrosion cast data, being the largest sample in the present study at the highest spatial resolution. Using these data, we can determine the angular insertion depth or insertion angle (*IA*) of an electrode as a function of the electrode insertion depth (*EID*) and the distance from the modiolus (*d*_*off*_).

We used this model to study the three currently most frequently used perimodiolar electrode arrays: the *Contour Advance electrode array* (CI612, Cochlear Ltd.), the *Mid-Scala electrode array* (HiFocus Mid-Scala, Advanced Bionics) and the *Slim Modiolar electrode array* (CI632, Cochlear Ltd.). These electrodes were all designed to come close to the modiolus and therefore modiolar variability is relevant for these implants. Furthermore, for all three electrodes, clinical insertion depths are available and can be compared to the outcomes of our estimations.

The analysis of the straight portion of the cochlear base and the critical diameters of the implant curvature was also performed based on this model. The potential location of the cochlear implants (red curve in Fig. 2C) was determined as a curve with an assumed constant offset (*d*_*off*_) to the wall of the scala tympani (dashed line in Fig. 2C). The three different values of *d*_*off*_ corresponding to the three types of precurved electrode arrays were calculated based on clinical findings on the respective ratios of metric and angular insertion depths. A more detailed description of how the model was used to derive the different values of *d*_off_ is given within the “Results” section. This allowed for the calculation of the array curvature *r*_pre_ necessary to achieve a specific insertion trajectory. The point of tangential transmission (*l*_*str*_ and *IA*_*str*_, respectively, Fig. 2C) was defined as the point where the tangent line to the position of the implant (dashed line) connects this point with the intersection of the A-axis and the lateral wall. This defined the angle of tangential transition *IA*_*str*_ and the straight distance *l*_*str*_. The distance *l*_crit_ represents to insertion depth at which a straightened array would hit the lateral wall and hence increase the risk of intracochlear damage.

Additionally, we studied the impact of modiolar variability on the risk of tip fold-over. In order to do so we introduced the critical radius *r*_fold_, describing the curvature of an array tip small enough to enable the array to “stand up” on the modiolar wall (i.e. the critical radius that allows for a 90° angle between array tip and modiolar wall, as is depicted in Fig. 2D; it is considered critical since an angle > 90° between array tip and modiolar wall will likely result in tip fold-over). Figure 2D shows that the critical radius *r*_fold_ is dependent on the individual morphology as well as on the angular insertion depth *IA*. Furthermore, the array will touch the lateral wall of standing up on the modiolar wall, i.e. the minimal distance between electrode array and the lateral wall *d*_LW_ needs to be taken into account. The critical radius was hence computed from IA = 90° (i.e. beyond the straight part of the electrode trajectory) to IA = 720° in 1° steps for each one of the 108 cochlear reconstructions. The exact value of *r*_fold_ was calculated as the radius of an arc (shown in red) whose one end stands up on the modiolar wall with a 90° angle while the opposite end merges tangentially into the path with an offset of *d*_LW_ off the lateral wall.

### Statistical analysis

The descriptive data are always shown as mean ± standard deviation. Statistical testing was always performed at α = 5%. Testing was performed in MatLab (version R2018a, The MathWorks Inc., USA) with two-tailed Wilcoxon–Mann–Whitney test when means were compared and Kolmogoroff–Smirnoff test when distributions were compared. When data were available only in the form of mean and standard deviation (from literature in the clinical data of Fig. 7), two-tailed t values were calculated manually from means, sample sizes and standard deviations and significance was determined from tabulated t values^47^. Pearson’s correlations (*r*) were used to analyze the relation between modiolar and lateral wall measures. As a measure of common factors of variability, r-values were squared and are provided in percent.

## Results

Using the large dataset of more than 200 human cochleae obtained with different methods, we first focused on measures that can be easily obtained in all these approaches. Using such strategy, it was possible to compare the different methods to each other and by that validate them.

**Figure 3:**
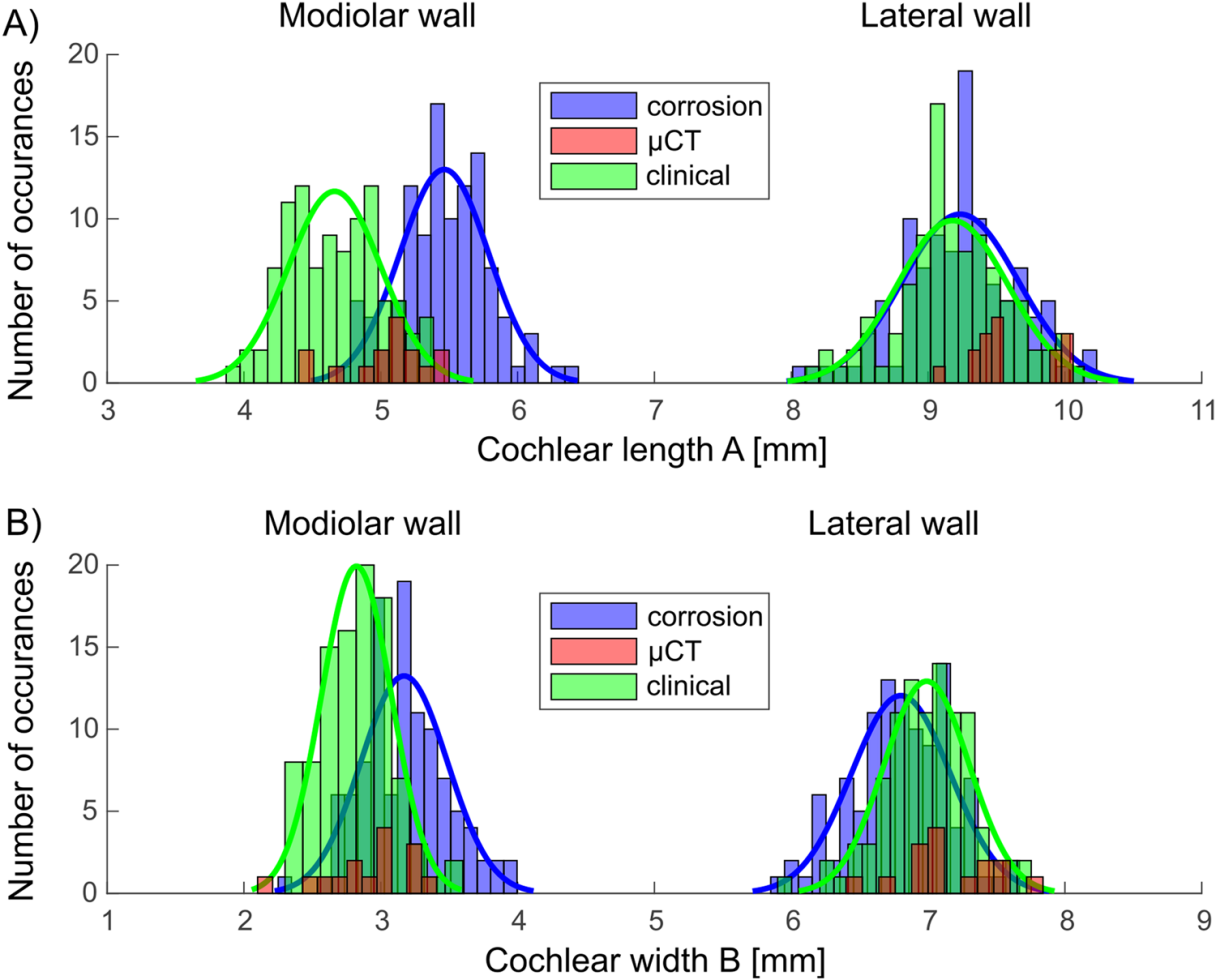
Variability of (**A,B**) measures of the lateral wall and modiolar wall in the three datasets used.

The most straightforward comparison of variability was using the measures obtained at A and B axes of the cochlea in clinical CTs, µCT and corrosion casts. Comparing the three methods reveals that all measures taken at the lateral wall are similar and overlapping with these techniques (Fig. 3). The differences were systematic at the modiolar wall and, for B-axis, also at the lateral wall (A-values lateral wall, mean ± standard deviation: corrosion 9.24 ± 0.42 mm; clinical 9.18 ± 0.40 mm, p = 0.2950; A-values, modiolar wall: corrosion 5.46 ± 0.32 mm; clinical 4.66 ± 0.34 mm, p = 1.9961 × 10^–29^, B-values, lateral wall: corrosion 6.80 ± 0.36 mm; clinical 6.99 ± 0.31 mm; p = 1.0996 × 10^–4^; B-values, modiolar wall: corrosion 3.17 ± 0.32 mm, clinical 2.82 ± 0.26 mm, p = 2.1310 × 10^–14^, two-tailed Wilcoxon–Mann–Whitney test). The measures taken with µCT were too few in number to well characterize a histogram. The individual datapoints, nonetheless, fall within the range observed with the other two methods (means for A-values, lateral wall: 9.60 ± 0.31 mm; modiolar wall: 5.04 ± 0.31 mm; B-values, lateral wall: 7.14 ± 0.34 mm; modiolar wall: 2.91 ± 0.32 mm).

The measurements demonstrated systematic differences in the methods. The corrosion casts had a larger A compared to the clinical measurements; the B-results were mixed. Particularly the modiolar clinical measures appeared systematically larger in the corrosion casts. This difference is likely given by the soft tissue at the cochlear base, since the measures taken with corrosion casts include soft tissue with the modiolar measurements, whereas the clinical CT and µCT visualize only the bone and exclude the soft tissue. These differences may have been further affected by the limited resolution of the clinical measurements. Most important for the present aim is, however, that the variance of the measures is similar for modiolar and lateral wall measures.

The coefficient of variation, relating the variance to the mean of the population and thus providing a quantification of the spread of the data, was nominally always larger, not smaller, for the modiolar measures (Table 1). This indicates that the interindividual variability of the modiolar wall is not smaller than the variability of the lateral wall.

**Table 1:** Coefficients of variations are consistently larger for modiolar measures compared to the lateral wall measures.

	Coefficient of variation			
	A measure		B measure	
	Lateral wall	Modiolar well	Lateral wall	Modiolar well
Clinical CT	0.0436	0.0730	0.0443	0.0922
Corrosion casts	0.0446	0.0586	0.0529	0.1009
μCT	0.0323	0.0615	0.0476	0.1100

**Figure 4:**
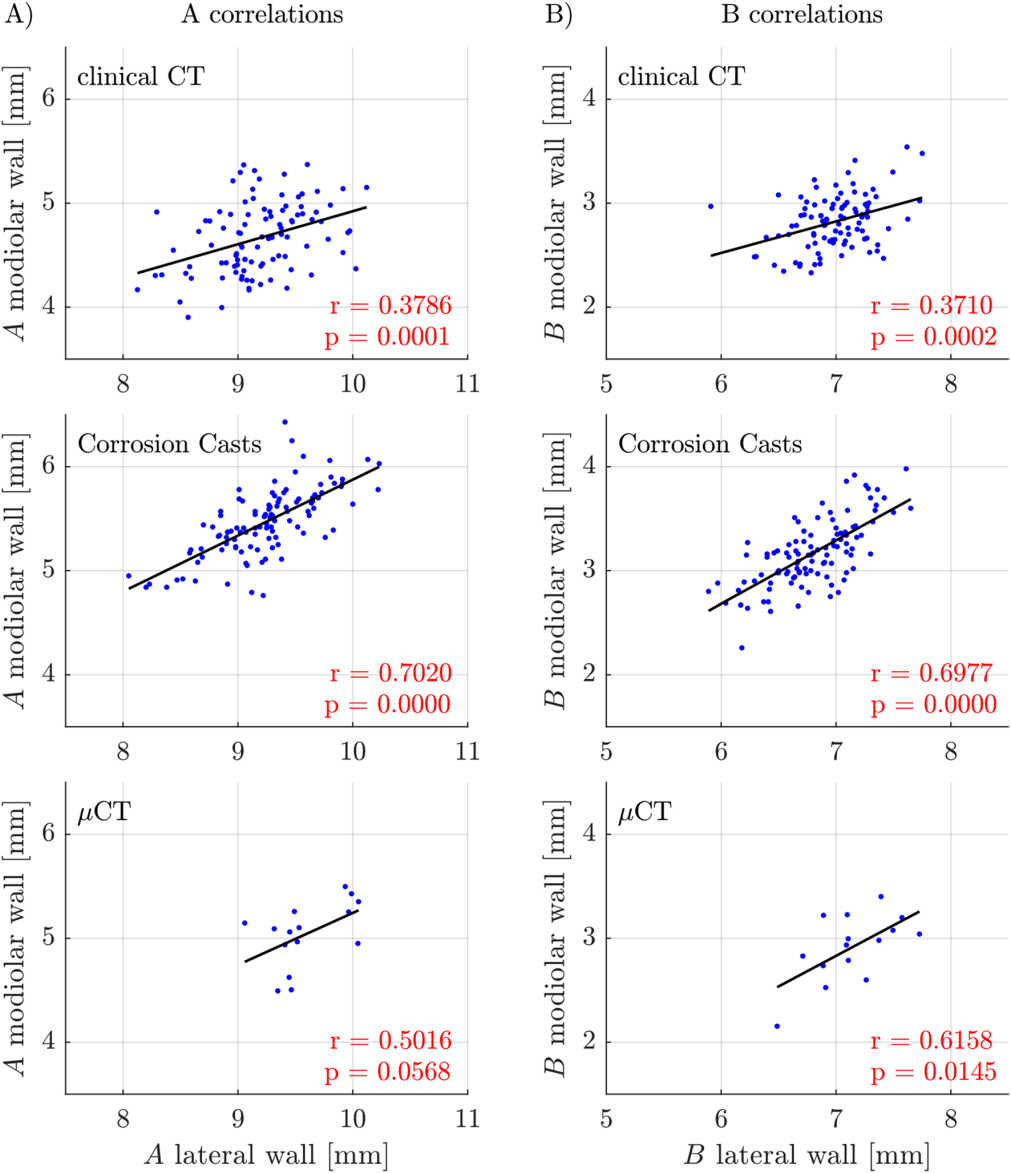
Correlations of (**A**) cochlear basal diameter A and (**B**) cochlear width B of the lateral and modiolar wall respectively, which were investigated for Clinical CT data (top row), Corrosion Casts (center row) and µCT (bottom row).

We subsequently analyzed the correlations between modiolar and lateral measures (Fig. 4). The values correlated significantly for all methods used. The best correlation was achieved for the corrosion casts (values of r ~ 0.7), where precision of measurement is likely highest (Fig. 4). Not unexpectedly this indicates that the measurements taken from clinical CTs are confounded by some measurement imprecisions due to low contrast and resolutions. Even in the few µCT measurements, the correlations were significant for the B values (Fig. 4B).

In the corrosion casts, the r^2^ suggests that approximately 49% of the variability of the modiolar measures was explained by lateral wall measures (and vice versa). This means that cochleae that are large in the lateral measures tend also to be large in the modiolar measures. However, there is also variability in the size of the cochlear spaces, contributing to the “noise” in this correlation and probably explaining the remaining 51% of variability.

**Figure 5:**
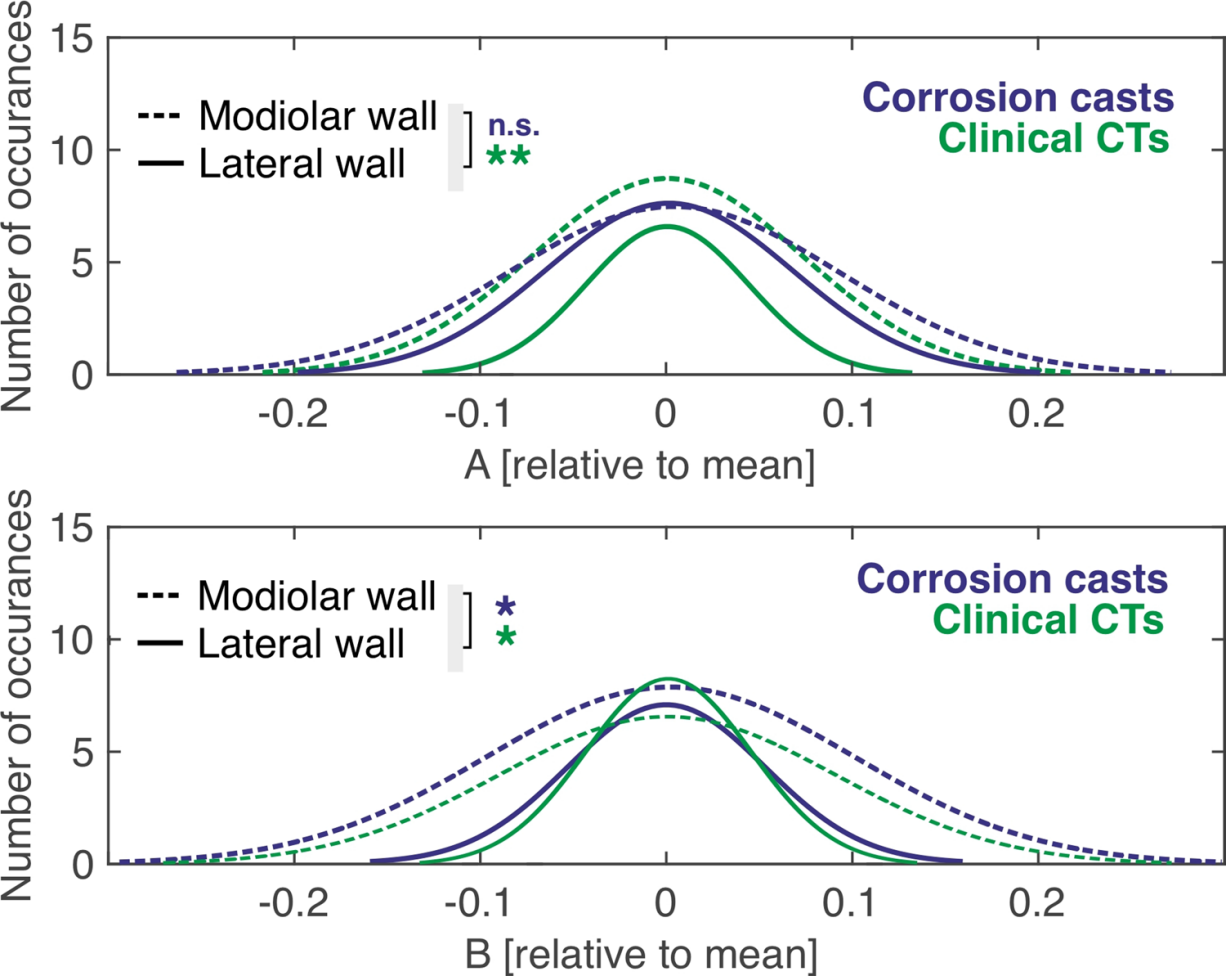
Comparison of the variance of lateral wall and modiolar wall measures after subtracting the mean and normalizing to the mean. *p < 0.05; **p < 0.01; *n.s.* not significant p > 0.05.

Given these results, we normalized the distributions (subtracted the mean and divided by the mean, see Eq. (1) so that modiolar and lateral wall measures could be overlaid and directly compared (Fig. 5). This confirmed the surprising result: here the modiolar measures had in part larger variance than the lateral wall measures (Kolmogoroff–Smirnoff two-tailed test, A values comparison, corrosion casts: p = 0.4939, clinical: p = 0.0073; B-values comparison, corrosion casts: p = 0.0429, clinical: p = 0.0118).

Finally, we also compared the measures between corrosion casts and the clinical CT measures: here the variance was not significantly different between the methods (modiolar wall A measures: p = 0.2438; B measures: p = 0.8527; lateral wall A measures: p = 0.8431; B measures: p = 0.4444; Kolmogoroff–Smirnoff two-tailed test).

**Figure 6:**
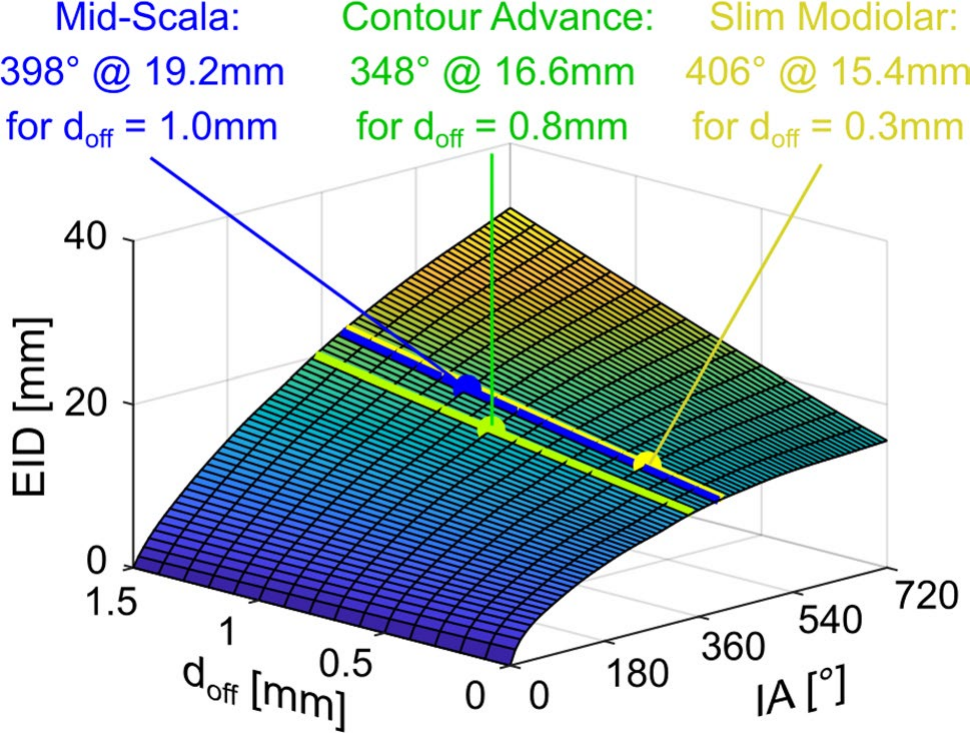
Dependency of the insertion depth (IED) to implantation angle (IA) on the distance from modiolus (d_off_) of three different commercial perimodiolar electrode arrays. Data approximated based on an individual corrosion cast reflecting the mean overall size of the human cochlea. For same implantation angle shorter insertion depth is required if the distance to the modiolus is smaller.

Subsequently, we tuned our insertion model (Fig. 2C) to the three different kinds of electrodes. As described in the methods, the model can be employed to compute the insertion angle (*IA*) dependent on the electrode insertion depth (EID) for a specific cochlea shape and distance from the modiolar wall (*d*_off_). Model tuning was hence done in the following manner: firstly, the average lateral and modiolar wall spirals were scaled to each one of the 108 corrosion cast datasets using the corresponding values of A and B. The insertion trajectory dependent on d_off_ could hence be computed for all individualized anatomies, which was done for different values of d_off_ ranging from 0 to 1.5 mm in 0.1 mm steps, yielding a total of 16 *EID*(*IA*) profiles for each one of the 108 anatomies. The EID(IA) profiles for a specific value of d_off_ were then averaged, and the resulting *d*_off_-dependent characteristics were combined into the three-dimensional profile depicted in Fig. 6, describing the average dependency of *EID*, *IA* and *d*_*off*_. The 3D profile shows that for more modiolarly located electrode arrays, as expected, smaller *EID*s are necessary to achieve specific *IA*s. Using clinical observations on the mean ratio of *EID* and *IA* for the respective electrodes, the electrode-dependent value of *d*_*off*_ could be derived: the mean profile showed an *IA* of 348° with an *EID* of 16.6 mm (as reported in Ref.^48^ for the Contour Advance) for *d*_*off*_ = 0.8 mm, an *IA* of 398° with an *EID* of 19.2 mm (as reported in Ref.^49^ for the Mid-Scala) for *d*_*off*_ = 1.0 mm and an *IA* of 406° with an *EID* of 15.4 mm (as reported in Ref.^50^ for the Slim Modiolar) for *d*_*off*_ = 0.3 mm.

**Figure 7:**
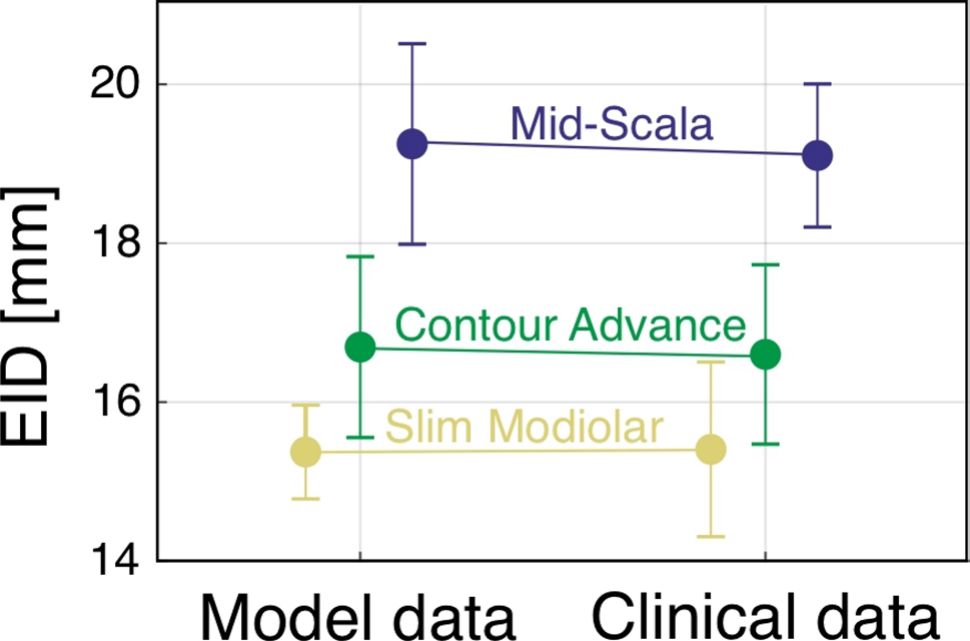
Comparison of model computations with previously published data on EID confirm the validity of the approximation based on corrosion casts, with nearly identical means and standard deviations. Clinical data for Contour Advance from Ref.^48^, Mid-Scala electrode from Ref.^49^ and Slim Modiolar from Ref.^50^. Differences were not significant in all comparisons (two-sided t-test, p > 0.05).

In order to validate if employing these offset values yields data on metric and angular insertion depth, which are comparable to clinical observations, we additionally took standard deviation data reported in the three publications on the respective perimodiolar arrays into account. Using the average shape of the modiolar wall, we used the model to compute the metric insertion depth (*EID*) necessary to achieve the reported average insertion angles ± 1 standard deviation of the respective electrode arrays. As shown in Fig. 7, the computed *EID* ranges necessary to achieve the clinically observed ranges of insertion angles are very similar to the ones assessed within clinical data: for the Contour Advance electrode the mean implantation angle of 348 ± 36° was clinically achieved with an EID of 16.6 ± 1.1 mm^48^ and the model prediction was nearly identical—with 16.7 ± 1.1 mm (p > 0.05, two-tailed t-test, Fig. 7). For the Mid Scala electrode, clinical data have shown that the mean implantation angle of 398 ± 41° required an EID of 19.1 ± 0.9 mm^49^ and the model prediction was again nearly identical—19.2 ± 1.3 mm (p > 0.05, two-tailed t-test, Fig. 7). For the Slim Modiolar electrode, clinical observations showed a mean insertion angle of 406 ± 33° with an EID of 15.4 ± 1.1 mm^50^ while the model predicted that these insertion angles can be achieved with an IED of 15.43 ± 0.06 mm (p > 0.05, two-tailed t-test, Fig. 7).

After this validation step, the model was used to investigate the insertions of perimodiolar arrays which follow the trajectories of commercial electrode arrays (due to the correspondingly matched *d*_off_ values of 0.3 mm, 0.8 mm and 1.0 mm) in more detail. This was performed by computing the relation of metric and angular insertion depths, i.e. what *EID* values are necessary to achieve specific *IA*s, for each one of the 108 cochleae with each one of the different values of *d*_off_. It is important to note that these results are theoretical predictions based on the electrode shape and the corrosion casts.

**Figure 8:**
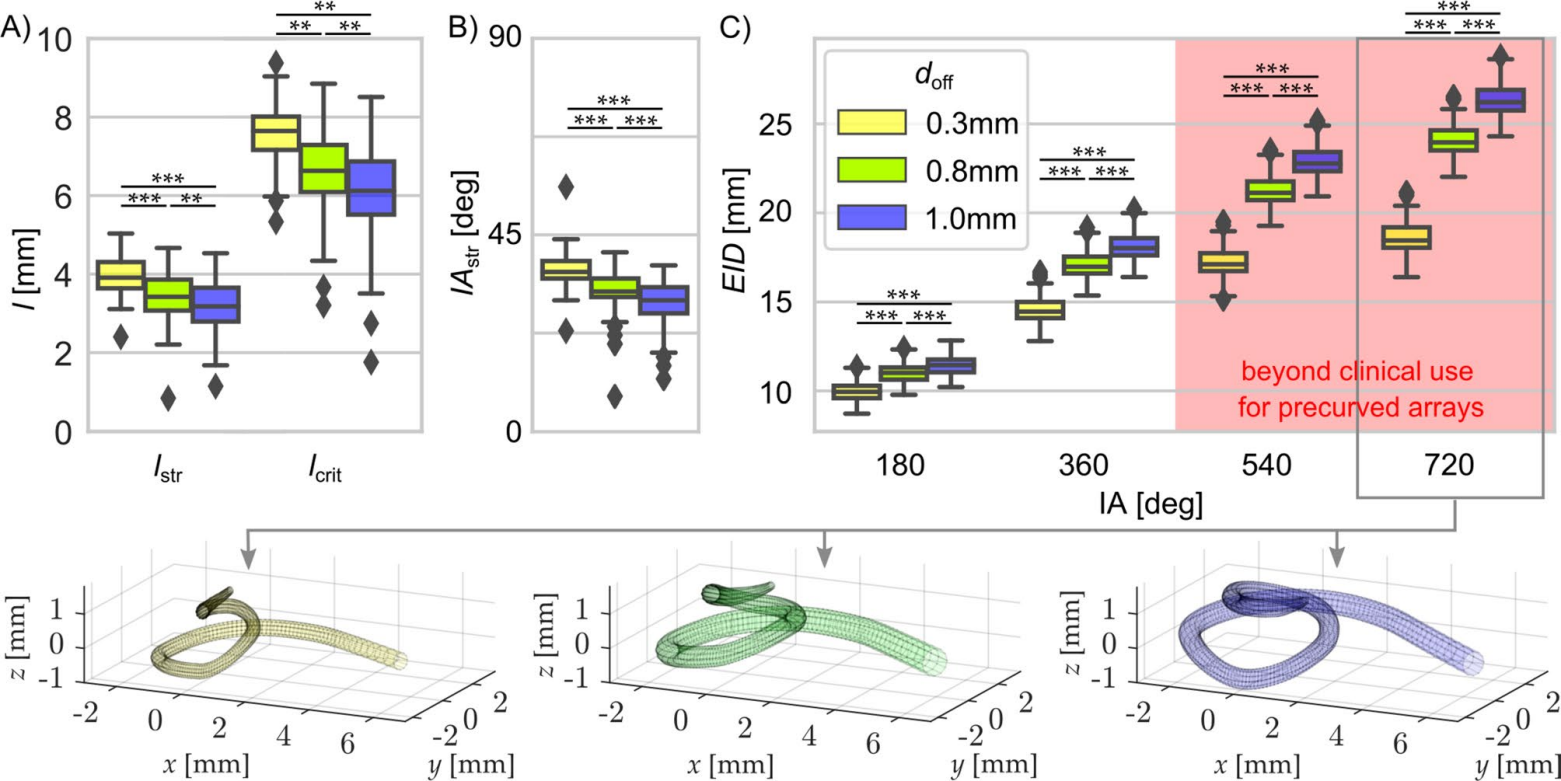
Approximated position of the cochlear implant array for three conventional perimodiolar electrodes with different distances *d *_*off*_ to the modiolar wall. (**A**) The straight portion of the implant trajectory *l*_*str*_ as well as the critical distance *l *_*crit*_ at which the straight portion would touch the lateral wall are largest for the electrode that is closest to the modiolus (in yellow). (**B**) Also, the implantation angle covered by the straight portion of the implantation *IA *_*str*_ is largest in the electrode that is closest to the modiolus. (**C**) Relation of insertion depth (EID, in mm) as a function of insertion angle (IA). Shown are theoretical values; perimodiolar or midscala arrays were not designed for the implantation of 540° or beyond. The electrode that is closest to the modiolus (*d*_*off*_ = 0.3mm) theoretically requires a shorter electrode array to reach the end of the second turn. The median trajectories for an insertion angle of 720° shown below suggest that close proximity to the modiolus (i.e. a small value of *d*_*off*_) requires a more complex array 3D curvature, which is likely to increase the risk of tip fold-over. Prisms designate outliers. The 21 statistical p-values are shown as asterisks, *p = 0.05; **p = 0.01; ***p = 0.001, two-tailed Wilcoxon–Mann–Whitney test.

The first critical measure of the insertion of perimodiolar arrays is the length of the straight portion of the implant in the basal cochlear turn, which should ideally correspond to the value of *l*_str_ depicted in Fig. 2C. However, this measure is highly variable and dependent on the position of the electrode array within the scala tympani. The distance *l*_str_ and angle *IA*_str_, after which the array passes the tangential point and thus may be safely released from its straightener (Fig. 8A+B), vary substantially for the electrode distance from the modiolus (*d*_*off*_). Thus, *l*_*str*_ and *IA*_*str*_ are strongly dependent on the individual cochlear anatomy. The same holds true for the distance *l*_crit_ after which the array would touch the lateral wall, potentially causing insertion trauma (if not yet released from the straightener). The results show that the three investigated offsets *d*_off_ result in different *l*_str,_ *IA*_str_ and *l*_crit_, i.e. all three parameters are not only dependent on the individual anatomy but also on the distance from the modiolus *d*_*off*_.

Interestingly, the ranges for the optimal release point *l*_*str*_ and the ranges critical for contacts with the lateral wall *l*_*crit*_ overlapped for *d*_*off*_ 0.8 and 1.0 mm. This demonstrates that for these distances from the modiolus there is no universally safe *l*_*str*_ that guarantees both (i) a safe release from straightener (without tip fold-over) and (ii) no risk of trauma at the lateral wall. In other words, there is no “value that fits all” and the surgeon’s guides for release from stylet require at least different values for small, mean and large cochleae. This highlights again the importance of individually assessing the patient anatomy prior to implantation.

Next, the interrelation of *EID* and *IA* was investigated for the different values of *d*_off_. The data, consistent with Fig. 6, further suggest that if an array is located closer to the modiolus, shorter insertion depths are required to achieve specific insertion angles (Fig. 8C). Modiolar electrodes of a certain length can thus theoretically achieve higher insertion angles than lateral wall electrodes of the same length. Pragmatically, these pre-curved electrodes are never inserted beyond or even up to 540°, which is most likely owed to the complexity of the insertion and trajectory the array must follow: the implantation with the stylet (in the straightened form) can only take place within the straight portion of the basal turn (*l*_*str*_). Afterwards the implant must be released and proceeds through the cochlea in its predetermined curvature, which, if not coinciding with the curvature of the cochlea it is inserted into, would increase the risk of tip fold-overs (which is investigated in more detail below). In order to highlight the increasing complexity of the necessary array trajectory for deep, perimodiolar insertions, the median trajectories for angular insertion depths of 720° are depicted underneath Fig. 8C. These suggest that especially for a very close proximity to the modiolus, the array needs to be very tightly twisted. In addition, the pre-curvature can no longer be two-dimensional but must incorporate the height change of the cochlear spiral. This further increases the risk of basilar membrane puncture in the base as the coiling force would likely be applied directly upwards against the membrane.

**Figure 9:**
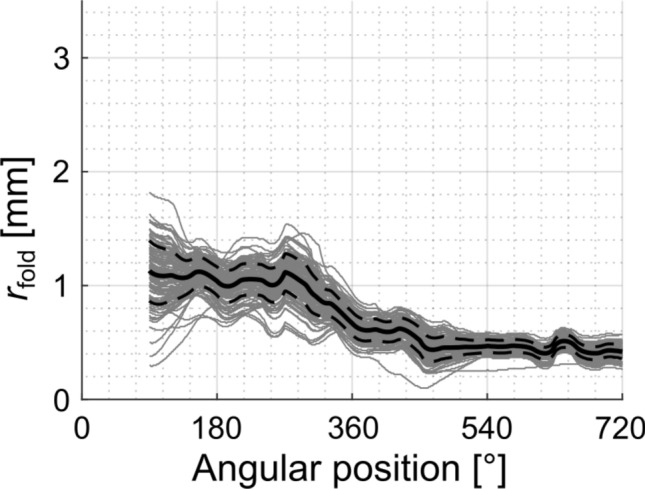
The critical radii (*r*_*fold*_) as determined from the 108 corrosion casts. The data reveal a rather flat function until 270°, with mean value of 1.13 mm and maximum values of up to 1.8 mm within the basal cochlear region. Around angular positions of 360°, the critical radii decline to < 0.7 mm.

In order to further quantify the risk of tip fold-overs, we analyzed the critical radii (i.e. the maximal curvatures of pre-shaped arrays that involve the risk of tip fold-over by exceeding the 90° angle to the modiolar wall) in more detail. For this, in each individual corrosion cast the critical radii *r*_*fold*_ (as defined in Fig. 2D) were determined between an insertion angle of *IA* = 90° (which is beyond the largest angle of tangential transition *IA*_str_ found within this study and hence always within the curved part of the electrode trajectory, cf. Fig. 8B) and *IA* = 720° (Fig. 9). These values were highly interindividually variable. Nonetheless, within the first 270° the critical radius functions were rather flat, with a maximum of the mean curve of 1.13 mm. This is of importance, since the release from the straightener (e.g. stylet in case of Contour Advance) must take place within the first 90°, but preferentially after the end of the straight portion of the implant course, thus after ~ 5 mm insertion (Fig. 8B). In consequence, to safely prevent tip fold-over at this position, the tip of the implant after release from the stylet should have a preformed radius ≥ 1.13 mm for the average cochlea such that the array tip cannot fold over within the basal cochlear region. However, the value of 1.13 mm is not optimal for all cochleae; to safely avoid tip fold-over in all cochleae, the radius should even exceed 1.8 mm.

**Figure 10:**
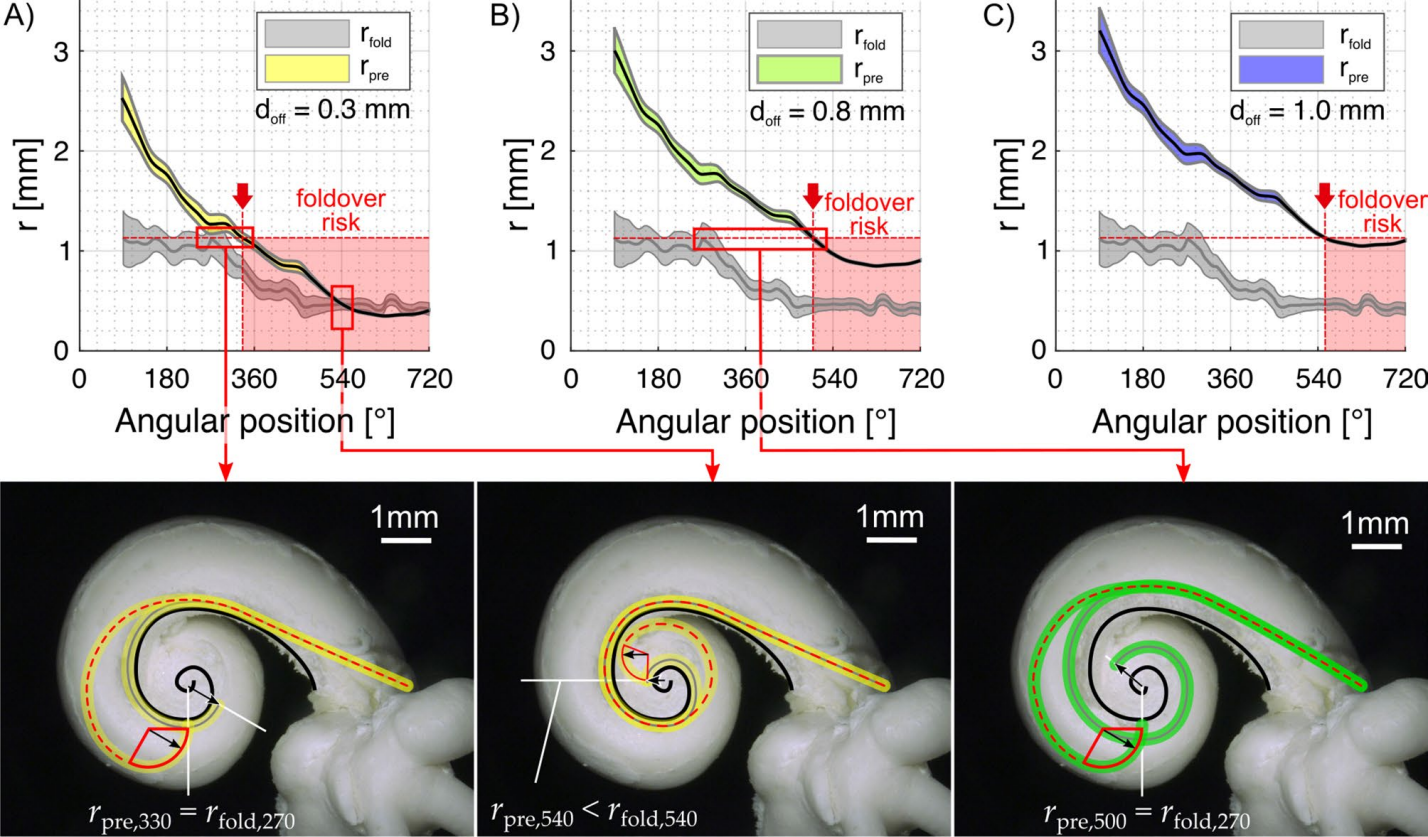
Mean (± standard deviation) of the radius (*r*_*pre*_, i.e. the curvature of the preformed implant, and *r*_*fold*_, i.e. the critical radius for tip fold-over, see Fig. 2) as a function of angular position from the round window for the three different designs of the implants, with three different assumed distances from the modiolus ((**A**) 0.3 mm; (**B**) 0.8 mm and (**C**) 1.0 mm). For comparison, mean values for the critical radius are shown in grey. Data obtained from corrosion casts. The red line depicts the maximal critical mean radius of 1.13 mm (occurring at about 270°). The red arrow points to the angular position at which this line intersects the individual optimal array curvatures. Beyond this point, this curvature would lead to an increased risk of fold-overs because it allows the array tip to buckle up on the modiolus (see Fig. 2A). The bottom images show examples of (from left to right) desired and critical curvature occurring at a similar angular location, the danger of the critical radius being even larger than the desired array radius and the desired curvature at an angle beyond 360° yielding an increased risk of tip fold-overs within the basal turn.

Since the modiolus becomes thinner in the apical direction, to come optimally close to the modiolus and remain closely positioned to the modiolus throughout the whole cochlea, the implant requires a particular radius (*r*_*pre*_) at each angular position. This curvature is dependent on the assumed distance of the array from the modiolus. The next question was if this characteristic of critical radii *r*_fold_ can be compared with the curvatures *r*_pre_ of different electrode arrays (cf. Fig. 2) to derive array specific statements on increased risks for tip fold-overs. We assessed these hypothetical best curvatures for the three above approximated distance values *d*_off_ of 0.3 mm, 0.8 mm and 1.0 mm, which correspond to the commercial electrode arrays Slim Modiolar, Contour Advance and Mid-Scala, respectively, up to the first quadrant of the second turn. Figure 10 hence shows the mean ± one standard deviation of the corresponding curvatures *r*_pre_ for which our model computes insertion angle comparable to clinical findings (Fig. 7). In addition, the mean profile of the critical radius *r*_fold_ ± one standard deviation as well as the maximum of the average critical radius of *r*_fold_ = 1.13 mm (dashed horizontal line) are displayed. Regarding the pre-curvature, all three array trajectories suggest decreasing *r*_pre_ profile (i.e. an increasing curvature) with increasing insertion angles as a consequence of the spiral profile of the cochlea with decreasing modiolar diameter. The different offsets *d*_off_, representing the different proximities to the modiolar wall, mainly create a vertical shift of this curvature profile. The consequence of this shift regarding the chance of tip fold-overs can now be derived if comparing the curvature profiles with the dashed horizontal line (representing the projection of the average critical radius *r*_fold_ in the cochlear base, occurring at about 270°, array independent) onto the array dependent curvature profiles. All 3 comparisons show an intersection of the dashed line with the curvature profiles, and the angular value at which this intersection occurs (red arrow) is of critical importance. When starting with the array with the smallest distance from modiolus (0.3 mm, depicted in Fig. 10A), the figure shows the intersection of the two curves at about 330° (red arrow in Fig. 10A), which lies within the range of clinically reported insertion angles with the Slim Modiolar array. This means that the tip curvature of this array necessary to achieve the desired perimodiolar location at 330° equals the curvature, which increases the likelihood of tip fold-overs at 270°. In other words, if releasing such a hypothetical array (designed so that its curvature fits optimally to the 380° point) from the straightener before or at the 270° point might yield a tip fold-over. The diagram in Fig. 10A further shows that after about 540°, the pre-curvature radius *r*_pre_ is even smaller than the fold-over critical radius *r*_fold_. Fold-overs beyond insertion angles of 540° are hence nearly inevitable with such array design. This demonstrates that for assuring atraumatic insertion without the risk of tip fold-over, the electrode should be designed to be located more than 0.3 mm away from the modiolus.

In contrast to the 0.3 mm array design, the curvature profiles of the two other investigated distances of 0.8 mm and 1.0 mm (Fig. 10B,C) do not show an intersection with the dashed line within the respective ranges of clinically reported insertion angles. Tip fold-overs can hence only be expected for cochleae with cross section larger than the average shape, which would yield a higher r_fold_ profile. This would result in an intersection with the pre-curvature profile of these arrays at lower angular positions.

It remains to be considered that mean *r*_*pre*_ values were used for the present considerations. However, these are highly variable between individuals, and only near the apex, the variability is less—as shown by the minimal standard deviation in Fig. 10 for the highest implantation angles.

## Discussion

The presented data provide evidence that the modiolar cochlear structures are either as variable as the cochlear lateral wall or, in some measures, even more variable than the lateral wall. In no case, the variability of the modiolar wall was less than that of the lateral wall. The interindividual variability of the human cochlea thus extends also into the modiolus that is, in contrast to the scalar spaces, primarily shaped by the early-developing neural structures.

The mechanistic explanation of cochlear variability has been so far based on the efficient packing hypothesis and the fact that scala vestibuli and scala tympani form after the differentiation of the surrounding neuronal structures. Since the present study did not assess neuronal structures directly, it cannot exclude the possibility that the neuronal structures are not variable and that only the scalar spaces approach them much closer in the smaller cochleae. This is, however, unlikely: the spiral ganglion is located extremely close to the scala tympani, the separation being only by a thin bony shell and sometimes a vessel (Fig. 9 of Ref.^51^ and Fig. 6 of Ref.^52^; see also Ref.^53^). Therefore, interindividual differences in the modiolar axes must involve variations in the 3D shape of spiral ganglion. This was in fact confirmed in a previous study where the metric length of the first two turns of the cochlea explained 83% of the variability of spiral ganglion length (Ref.^7^, see also^53,54^). This information is key for surgical planning and an estimation of cochlear position to the individualized cochlear characteristic frequency^53^ may be used for a prediction of the individual cochlear frequency map, as incorporated in a first 3D model recently^46^. This may further help to provide more physiologic electrode-frequency allocation for programming of the CI processors. Most likely, it is already early in development when this part of the variability is established, i.e. before the scalar spaces appear. This suggests another inherent source of variability of the cochlear size, potentially related to the overall size of the temporal bone that is additional to the efficient packing.

Methodologically, when comparing the lateral wall and the modiolar wall we need to consider that the borders of the lateral wall are much better defined in all imaging techniques. The modiolar wall is fenestrated, and thus the border is harder to identify than the lateral wall (Fig. 1). One can assume that the outcomes of modiolar measurements will be more affected by measurement imprecisions (noise) than at the lateral wall. This may have substantially contributed to the larger spread of the data for the normalized modiolar distributions compared to lateral wall (Fig. 4). The interesting finding is, however, the high correlation (r ~ 0.7) of both measures in corrosion casts (with the best spatial resolution, Fig. 3A+B). This demonstrates that the results in corrosion casts are not driven by measurement “noise” (that would be uncorrelated), but rather by true variability behind the data. Such common factors explain 49% of the variability of lateral and modiolar dimensions. Of key importance is the use of several techniques: here clinical CT was much more contaminated by such uncorrelated noise, and consequently the r values were smaller, ~ 0.37. Interestingly, where measurements can be performed exactly, in µCT, despite few data, correlation coefficients are higher than in clinical CTs (Fig. 4).

The modiolar A and B values were smaller in clinical CT than in corrosion casts, most prominently for measure A, but observable also for B. The µCT measurements were positioned in between. The CT measures reflect the bony structures and exclude soft tissue near the modiolus and the lateral wall, whereas the corrosion casts, in fact, show only the empty spaces and as a negative image include, particularly in the modiolar measures, the soft tissue. Additionally to the imprecisions in the assessment of the modiolar wall, this may further contribute to these differences.

### Clinical implications

We investigated the consequence of the modiolar variability on the cochlear implantation. We have focused on three arrays that cover a wide range of distances from the modiolus. If comparing the present data on the ratio of metric and angular insertion depth of the perimodiolar arrays to data on straight electrode arrays, it becomes evident that perimodiolar implants of the same length have the potential to reach deeper into the cochlea. Avallone et al.^55^, for instance, found that with straight arrays, approximately 26 mm insertion depth are necessary to achieve insertion angles of about 540°. This length lies several millimeters above the lengths derived for all of the perimodiolar arrays within the current study (cf. Fig. 8). However, the use of the latter includes risks in cochlear trauma and comes at a cost of a complex design that currently does not allow deep implantation (see also below): since the implant must be preformed, implantations require a stylet (or straightener).

Furthermore, perimodiolar arrays require a precurved geometry. Precurved electrode arrays often have a constant curvature along the array—in other words, they are optimally designed for one insertion position (*r*_*pre*_ curves in Fig. 10). Basally to this position, the curvature will be smaller than optimal. Beyond this point (apical to it) it will be too large and thus come to lie further abmodiolarly, at an intermediate position between the modiolar and the lateral wall (comp.^56^).

Two additional anatomical limiting factors for perimodiolar electrodes require consideration:

1. The acceptable straight portion of implant course varied in different cochleae. The individual optimal straight insertion depth covers a range from 2 to 5 mm (Fig. 8B) depending on the microanatomy of the individual cochlea and the array to be implanted. The straightener itself can cause a cochlear trauma if inserted so deeply into the cochlea that it hits the lateral wall. The range of distances from round window straight to the lateral wall (*l*_*crit*,_ along the course of *l*_*str*_ in Fig. 2) in the present study was 3.5–9.37 mm. The surgeon’s guide for the Contour Advance electrode informs that the electrode tip is 7.6 mm from the marker for optimal insertion. For the Slim-Modiolar electrode array the literature provides the information of “about 5 mm” insertion before straightener removal^57^ and the Surgeon’s guide for the Mid-Scala gives 5.4 mm (distance between marker and tip of the electrode). These surgical recommendations lie beyond the point where the straight electrode array passes the modiolus tangentially, as is shown in Fig. 8A, meaningful for a safe release from straightener. It appears, however, that these recommendations may risk a contact between the straightened array tip and the lateral wall for many of the cochleae investigated in this study (see also^58^). Knowledge of the size of the straight distance (*l*_*str*_) and the maximum length till lateral wall is touched allows for individualizing the implantation procedure; however, due to resolution of clinical CTs, use of cochlear models may be needed for assessing this parameter precisely^46^.
2. The diameter of the modiolus decreases in the apical direction. The precurved diameter is dependent on the point where the release of the array from the stylet takes place (Fig. 10). The deeper the implantation, the smaller the diameter. At present, perimodiolar implants are mainly designed for implantation into the first turn. Nonetheless, higher cochlear coverage may provide more independent information channels and thus better speech understanding^16,59^. Thus, perimodiolar arrays always trade optimal position and risk of tip fold-over.

The preformed implant should consider that apically the diameter of the curvature must be small to adhere to the modiolus in apical portions of the cochlea. This, however, may lead to tip fold-over if the release is taking place at the end of the straight portion of the implantation (after <45° implantation angle, Figs. 2, 8C, 9), where the critical radius *r*_*fold*_ is nearly identical to the hypothetical optimal curvature of the array tip. To prevent tip fold-over in this region, the preformed radius should exceed 1.13 mm. This, however, is larger than e.g. the curling radius of the Contour Advance electrode array^60^. The Contour Advance, likely in the intention to avoid this, has a conic straight silicone tip that extends for ~ 1 mm and is not curved. This is probably intended to lean on the modiolus and prevent a fold-over. Nonetheless, even experienced surgeons cannot prevent tip fold-over in all cochleae with this electrode^21,33,34^, indicating that this approach is not always successful.

This critical radius *r*_*fold*_ is too large for the more apical portions of the cochlea, where such curvature would again move the tip of the implant array away from the modiolus. This is in fact also observable in clinical analyses of the location of the cochlear implant in the human cochlea with modiolar-close and -distant portions of the array depending on the angular position^38,61^. Our data suggest that particularly implantations > 500° would show the effect—the present day perimodiolar electrodes do not penetrate beyond this point.

Furthermore, at the border of the first and the second turn also a critical point of the vertical profile is observed in half of the cochleae (a vertical jump^7^) that might further complicate such implantation. However, in perimodiolar positions the vertical profile was much smoother than in the lateral positions^7^.

To optimize the implantation procedure and to exclude the risk of a tip fold-over, the present days electrode designs should aim at a distance to the modiolus of > 0.3 mm or provide larger curvatures (> 1.13 mm, best > 1.8 mm) after release from the straightener/stylet (Fig. 10). Clinical imaging outcomes of electrode array in use within the first cochlear turn show distances in the range 0.60–1.67 mm (for Cochlear 532/632 array 0.80 ± 0.10 mm and for 512 array 0.76 ± 0.07 mm; data from Ref.^62^). Closer locations, and thus true “modiolar hugging electrodes”, particularly those aiming at implantations beyond 400°, require new surgical and technical approaches due to the changing diameter of the modiolus. Only electrodes that are implanted more laterally and subsequently approach the modiolus slowly, after the implant has been placed (e.g. by the increased temperature in the inner ear in implants integrating temperature-sensitive memory materials^63^) represent a viable approach for true modiolar-hugging electrodes extending beyond the first turn of the cochlea. Here, however, the approach to the modiolus should start basally and continue later apically to prevent that the implant is dragged out of the cochlea (which would occur if the process was opposite). Such approach may, however, involve a significant force on the modiolus, with associated risk of trauma. It is worth further investigations, given that modiolus-hugging electrodes in the past provided such excellent channel separation (in some patients) that multi-channel compressed analogue stimulation (providing temporal fine structure) could be clinically used^64^. Similarly, some studies indicate better speech perception with perimodiolar electrodes^65^.

An interesting suggestion for achieving a better modiolar hugging position in the basal portion of the cochlea with current design of perimodiolar arrays is the “pull-back” technique^66,67^: after full insertion of the perimodiolar array the electrode is retracted back to eliminate buckling from the modiolus in the base. This might assure a better positioning in the base and does reduce the spread of excitation^66^.

Finally, the modiolar variability underscores the surgical challenges in trauma-free and fold-over-free implantations of perimodiolar arrays. The study strongly emphasizes the need of individualized implantation procedures for these arrays, with cochlear imaging and detailed planning using all methods available, including 3D cochlear models^46^. In a follow-up study, we are currently integrating the previous model of the lateral wall variability with the modiolar simulations to provide a unified tool to the clinical community. The most recent version of our model can be found on our website (https://www.neuroprostheses.com/AK/CochleaModel.html).

### Cochlear variability beyond efficient packing

The present results also provide deeper understanding of the cochlear microanatomical variability and its reasons. Differences were noted in the extent of variability between A and B measures of the modiolus. Similarly, also in a previous study this has been described and has been interpreted as the facial nerve having a larger effect on the B axis of the cochlea compared to the internal carotid’s effect on the A axis (Supplementary Fig. 4 in Ref.^8^). Since modiolar variability is in fact larger than lateral wall variability, this suggests the action of at least two different factors.

While the present data are largely consistent with the efficient packing hypothesis^8^, they call for an extension of the previous theory. We suggest the action of three independent factors in cochlear variability:

1. *Inherent variability* of the overall size of the cochlea affecting both the modiolar variability and lateral wall variability, presumably a genetically inherited factor. Both the A and B measures correlated with r^2^ = 0.64^8^, and modiolar and lateral wall measures correlated similarly (r^2^ = 0.49; present data). This together suggests that inherent variability is responsible for the common ~ 50% of the interindividual variations in all these measures and that it acts as a common background for all variations. It may be the size of the petrous bone that affects the overall size of the cochlea and is well observable in modiolar variability of B measure. This factor thus genetically “programs” the cochlea to “grow larger”.
2. *Limiting factor* of neighboring structures, particularly facial nerve, as observed previously^8^, is the second key player, potentially explaining the large part of the remaining variation (1 − r^2^ = 0.51). The action of this factor is stronger in extend at the B axis, where the closest structure, the facial nerve, is found. Proximity of the facial nerve limits the inherent variability of the lateral wall and causes this variability to be smaller than the modiolar variability. Limiting factors affect the growth involved in the inherent variability in some cochleae by preventing it “growing larger” along a specified direction. Such factors would be responsible for the complex, irregular geometry of the cochlea including dips, indentations and jumps in the form, as reported previously more prominently along the lateral wall^7,8^.
3. *Measurement noise* that constitutes a part of the 51% mentioned in the *limiting factor* above. For modiolar wall, this imprecision is larger than for the lateral wall, the extent of it is, however, not clear yet.

These considerations suggest that the full understanding of the mechanisms of cochlear interindividual variability requires, additionally to understanding of limiting factors^8^, also the elucidation of the inherent factors likely driven by genetics.
